# (Co/Zn) Al_2_O_4_ nano catalyst for waste cooking oil catalytic cracking

**DOI:** 10.1038/s41598-022-10596-z

**Published:** 2022-04-23

**Authors:** R. El-Araby, M. A. Ibrahim, Elham Abdelkader, E. H. Ismail

**Affiliations:** 1 Chemical Engineering and Pilot Plant Department, National Research Centre, Cairo, Egypt; 2 Chemistry Department, Ain Shams University Chemistry Faculty of Science, Cairo, Egypt

**Keywords:** Energy science and technology, Engineering

## Abstract

The current work investigated the preparation of Nano-particles of Co/Zn Al_2_O_4_ as a catalyst via co-precipitation method. Several analyses, including BET, XRD, HRTEM, EDX, SEM, and FTIR, were used to characterize it. The analysis revealed that the prepared catalyst had an average surface area of 69.20 m^2^/g, a cross-sectional area of 16.2 m^2^/molecule, an average particle size of approximately 28 nm, and a pore size of 0.22 cm^3^/g. The prepared catalyst was used in a bio fuel synthesis process via thermo-catalytic cracking of waste cooking oil (WCO) in a single step batch reactor. Catalyst loading was tested with different weight percentage of 1.5%, 2%, and 2.5%. The pilot study revealed that the best conditions for optimizing bio jet fuel yield were 400 °C, a catalyst loading of 2%, and a reaction time of 30 min.The optimal cut-off from the distillation process of crude liquid bio fuel product which represents a fraction of bio-jet fuel was in the range from 150 to 240 °C.

## Introduction

The need for raw materials and energy is increasing globally due to the increase in the population and the standard of living. Therefore, there is a growing in the use of fossil fuels, which leads to resorting to waste treatment in an attempt to provide a source of competitive and sustainable energy that reduces dependence on fossil fuels and is more environmentally friendly as it is less in emissions^1, 2^.

One of the most important fuels obtained by processing and upgrading a number of biomass and raw materials for degradable municipal waste is what is known as transportation fuel^3, 4^.

Tadios and Yedilfana used oil derived from the Scenedesmus algal specie to trans esterify biodiesel using a Nano catalyst made from goat bone debris with an average particle size of 43.96 nm. The Nano catalyst has a high concentration of active sites, with an average of more than 65 percent calcium oxide^5^. Recycling WCO for biofuel production in the presence of a catalyst has several advantages, including economic, environmental, and waste management benefits^6, 7^.

Yeshimebet et al.produced 94% biodiesel from low-cost waste cooking oil utilizing an improved CaO nano-catalyst made from chicken eggshell by hydration-dehydration treatment followed by calcination. The reaction parameters were as follows: a 1:12 oil to methanol molar ratio, a 2.5 wt percent catalyst loading, 60 °C, and a 120-min reaction time^8^.

While The thermal decomposition technique was employed by Yeshimebet with his team to create a CaO nano-catalyst with a mean particle size of 29 nm, which was then used as a catalyst for biodiesel generation in the transesterification process from WCO^9^.

The liquid organic products that result from the breakdown of triglycerides are considered to be a fuel with excellent distillation properties compared to petroleum fuels^10, 11^. Therefore, some studies used distillation in the separation of the cracked products, and specific cuts were analyzed^12, 13^.

This boiling range's hydrocarbon constituents have 4 to 12 carbon atoms in their molecular structure and are classified into three types: paraffins (including cycloparaffins and branched materials), olefins, and aromatics. Naphtha is a catch-all term for the low-boiling hydrocarbon fractions that make up a large portion of gasoline. for low-boiling hydrocarbon fractions that make up a significant portion of gasoline. Aliphatic naphthas are those that contain less than 0.1 percent benzene and have carbon numbers ranging from C3 to C16. Aromatic naphthas have carbon numbers ranging from C6 to C16 and contain high levels of aromatic hydrocarbons such as benzene (> 0.1%), toluene, and xylene. Kerosene, also known as paraffin or paraffin oil, is a flammable liquid^14, 15^.

The scientist (Numez) conducted the initial investigation of the vegetable oil hydrocracking process in 1984, employing Rhodium and Ruthenium as catalysts for the hydrocracking of soybean oil in a single batch reactor^16^. Then, two years later, the scientist Numez and his team studied the hydrocracking of soybean oil and explained that the processes of decarbonylation and decarboxylation of fatty acids begin at temperatures of 400 °C using various types of catalysts to obtain short chain hydrocarbons such as paraffins and olefins, and the best of them was Zeolite because its Strong acidity aids in the hydro isomerization process to produce the hydrocarbon^17^.

In 2005, Charusin stated that in order to achieve the greatest results in the catalytic hydrocracking process, the reaction time must be increased for more than 90 min as temperatures rise. Then, in 2006, Charusin investigated the catalytic cracking of WCO at temperatures ranging from 280 to 430 °C and hydrogen pressures ranging from 1 to 2 bar over a reaction time of 45 to 90 min using Zeolite (HZSM-5), Hybride catalyst HZSM-5 Sulfated Zirconium, and Sulfated Zirconium catalysts. According to the results, the hybrid catalyst generated the best results in terms of producing gasoline fuel^18^.

In 2009, Nasikin obtained bio-gasoline from palm oil using hydro-catalytic cracking and a batch reactor in the presence of hydrogen at temperatures of 300 and 320 °C and a reaction time of two hours, with NiMO Zeolite) as a catalyst. Bio-gasoline was obtained from (C8–C10) by approximately 12%, and bio-diesel by 13%^19^.

In 2011, the scientist (Tiwari) reported that bio-gasoline is produced using (NiMO/Zeolite) at temperatures ranging from 300 to 320 °C in a batch reactor for 1 to 2 h, because Zeolite stimulates the production of a large amount of gasoline from palm oil due to its superior ability in cracking reactions^20^.

In other investigations, the goal has been to produce liquid organic products containing hydrocarbons in the gasoline range^18, 19^, as well as hydrocarbons in the aviation kerosene range, and in certain cases, hydrocarbons in the diesel range^18–20^.

The reactions that take place in the hydro processing are consisted of two groups: hydrocracking and hydrotreating. During hydrocracking process, a destructive hydrogenation takes places to convert higher molecular weight components to light products. While, isomerization and cracking of C–C bonds occur in slightly larger molecules in order to produce hydrocarbons in the boiling range of gasoline and diesel. An elevated temperature as well as pressure of hydrogen is used in this treatment to reduce the reactions of the condensation chain polymerization, leading to the formation of coke^20–22^.

Catalytic cracking is considered one of the promising technologies for preparing biofuels due to its simple operations and the similarity of the resulting fuel to petroleum fuels. Therefore, catalytic cracking can be used to produce hydrocarbon in range of gasoline, kerosene and diesel in the presence of catalysts, and it offers a very good suitability with a variety of raw materials, as well as a relatively lower cost. And in order to reach the requirements of fuel standards for the transportation vehicle, catalytic cracking still needs to decrease the acidic value and oxygen content of biofuels to improve the properties of the fuel such as density, calorific value, dynamic viscosity^23, 24^.

In the past decades, variety of catalysts, including solid-basic and generally acid catalysts were used in order to obtain the desired products. Studies have been conducted on the use of Na_2_CO_3_ or K_2_CO_3_ as primary catalysts for catalytic cracking of soybean oil and crude palm oil in the production of biofuels^25, 26^. The results indicated that the conventional basic catalysts could reduce the acid value effectively. The acidic solid catalysts such as Al_2_O_3_, HZSM-5, and MCM 41 and their effects on the catalytic cracking of lipids for biofuels have been extensively studied^27, 28^. There are two main advantages for the addition of the catalyst. First one is to accelerate the rate of the reaction and the second one is the active selectivity for specific products^29–31^. The temperature needed for catalytic cracking is (450° C), which is significantly lower than pyrolysis temperature of hydrocarbon (500–850° C) as reported in several studies^26, 27^. In a laboratory reactor at 450° C and 1–5 atm pressure, several studies have been reported that palm oil could be cracked using many different catalysts. The outcome hydrocarbon products were in rang of gasoline, kerosene and diesel fuel, with main amount yield of gasoline^32, 33^.

In a previous study, cobalt-alumina catalysts with high carbon loading (35–70% by weight) were synthesized by co-deposition. The effect of activation (reducing) temperature and CO_2_ loading on its efficiency in green diesel production was studied by selective deoxygenation (SDO) of solvent-free (SO) sunflower oil and cooking oil (WCO)^34^. In another study, zinc aluminate (ZnAl_2_O_4_) was used as a catalyst and proved effective in reducing the H/C ratio, which indicated that the product of thermo-catalytic cracking oil via ZnAl_2_O_4_ was not only an aromatic compound, but more saturated alkanes^35^.

The contribution of this work is to prepare and characterize (Co/Zn) Al_2_O_4_ nanoparticles, study their effect as a catalyst in the thermo-catalytic cracking of waste cooking oil in a batch reactor without using hydrogen, then subject the final product to fractional distillation process to separate the bio jet fraction range. Also, the aim of this work is to investigate the effect of various process parameters on biofuel yield%, such as temperature, catalyst loading, reaction time. Subsequently, prepare blends of this fraction with commercial Jet-A in various ratios and eventually, test the characteristics of the blends such as flash point, gum content, and freezing point to verify their agreement with the international specifications of ASTM for jet fuel.

## Experimental

### Materials and methods

#### Waste cooking oil preparation

Waste cooking oil collected from local fast food restaurants. The stage of preparing the oil begins with filtering through two steps: First, through a coarse sieve to get rid of the solid content. Then the filtration process is carried out through filter paper to ensure the removal of solid impurities. Then the waste cooking oil is kept in a container at room temperature. Prior to analysis and treatment, the oil is heated and stirred for two hours at 110 °C to remove any moisture.

The mass lost in the filtration and drying stage does not exceed 5% of the mass of the crude WCO.

#### Catalyst synthesis

**Figure 1:**
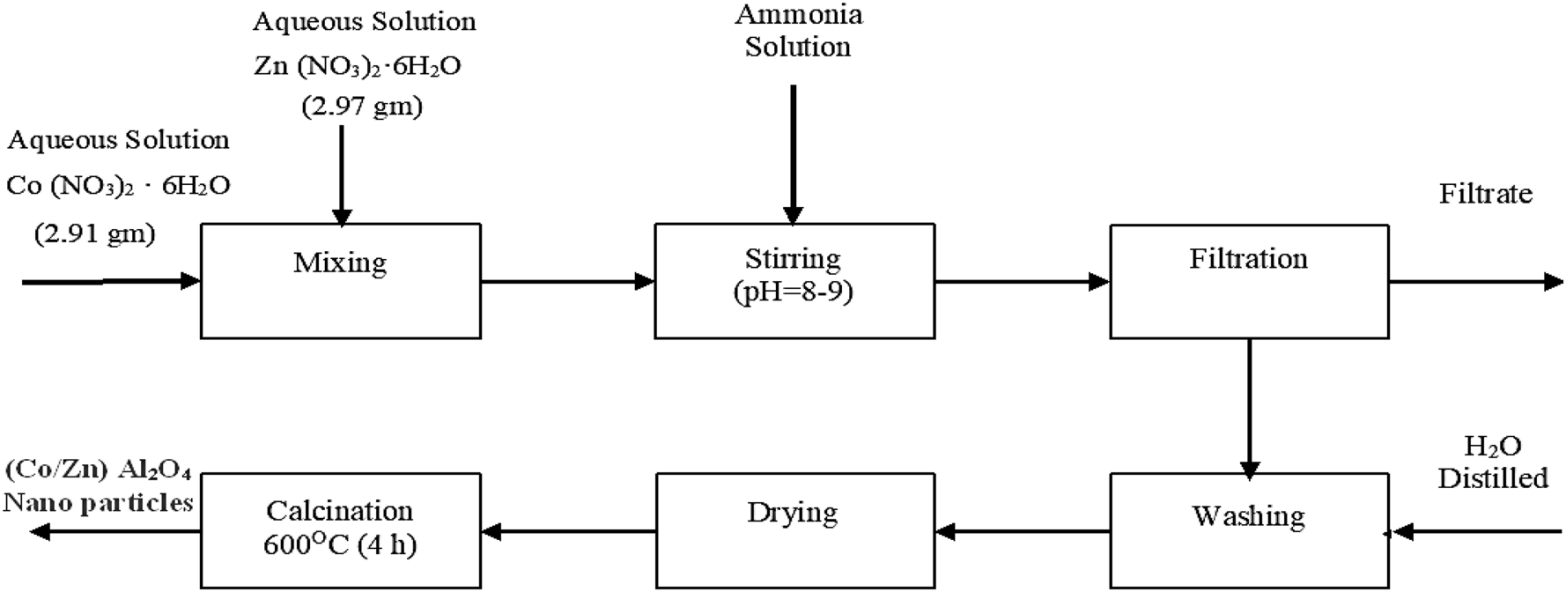
Catalyst synthesis process.

Co-precipitation method^36^ has been used to prepare cobalt zinc aluminate nanoparticles in an aqueous solution from metal nitrates using ammonia as a precipitating agent as shown in Fig. 1. Firstly, Zn (NO_3_)_2_.6H_2_O (10 mmol) (2.97 gm) and Co (NO_3_)_2_.6H_2_O (10 mmol) (2.91 gm) dissolved in 10 mL of distilled water were added to a solution of Al (NO_3_)_3_.9H_2_O (40 mmol) (15.005 gm) in 10 mL of distilled water. Secondly, a suitable amount of aqueous ammonia solution (25 wt%) was added to the above solution. The mixture was then thoroughly mixed until a complete precipitation at a pH between 8 and 9 was obtained. The precipitate that formed was filtered, washed with distilled water, and dried. Finally, the dry precipitate was calcined for 4 h at 600 C to produce (Co/Zn) Al_2_O_4_ nanoparticles.

#### Thermo-catalytic cracking process

A stainless-steel batch reactor operating under pressure with a capacity of 400 ml was used to run the experimental work. The pressure is measured by means of a pressure gage indicator and heating is carried out by means of an electrically operated heating jacket. The reactor internal temperature has been controlled by an Integrated Derivative Panel (IDP). The reactor vessel is loaded with oil (100 ml) and the amount of (Co/Zn) Al_2_O_4_ catalyst, nitrogen pressure starting at 5 atm, then pressure increasing with temperature and time. The operating conditions were altered as follows:

- The time is from 10 to 30 min.
- Catalyst concentration of 1.5 to 2.5 wt.% of WCO.
- The temperature is from 300 to 450 degrees Celsius.

#### Fractional distillation

The atmospheric distillation method ASTM D-86 is used for petroleum products and middle distillates. This method detects the boiling range for lots of different hydrocarbon distillates such as gasoline with or without oxygenates, diesel, and other light and middle distillates^37, 38^.

The collected crude liquid product from the thermo-catalytic cracking was centrifuged to split up any suspended particles. After that, it was subjected to a fractional distillation process to obtain fractions over a different boiling point temperature ranges from about 50 °C to about 350 °C. Percentage volumes of these fractions were calculated for bio gasoline at a boiling point temperature range of (60 °C–170 °C), bio jet fuel at a temperature range of (170 °C-270°C) and biodiesel at a temperature range of (270 °C-330°C)^39, 40^.

#### Catalytic activity

The conversion of the WCO into gases, liquid product at the end of reaction is relate to a catalytic activity which is dependent on the adsorption of the reactants on the active sites of the catalyst surface. Chemical adsorption is the main factor controlling the activity of catalysts.

The percentage yield of the WCO regarding to (x) product during the reaction is calculated by the following Eq. (1)^41^^41^:

1 $${\text{\% Yield }} = \frac{{\text{P }}}{{\text{F }}}*100$$

where P is the product weight and F is the input feedstock of WCO weight.

#### Characterization of WCO

The main physical and chemical characteristics of the raw waste cooking oil were determined. The fatty acid composition of the WCO was analyzed by GC with capillary column DB-5(60 m: ID 0.33 mm). Helium was used as a carrier gas at flow rate 1 ml/min, column temperature was kept at 240 °C–143 °C for 30 min. The density of fluids is measured using hydrometers. It is a glass laboratory floating body with a cylindrical stem and a bulb containing a metal weight. The density value is derived directly from the reading scale at the top section of the glass body with its measuring units, depending on the depth that the sample is plunged into. A fluid's viscosity is a property that measures its resistance to flow. A laboratory "DV-II + Pro" viscometer is used to measure the sample, which calculates the viscosity of a specific fluid based on shear stress and shear rates based on specific liquid temperature and drive motor rpm. The flash point of an oil, or the flash point of any fuel, is the lowest temperature at which liquid fumes will ignite in the presence of an ignition source. The ASTM specification calls for the PENSKY MARTENS FLASH POINT TESTER to be used to determine the flash point of fuels. The sample is kept in an open/closed cup that is heated at different rates. A flame is projected onto the liquid's surface from a suitable height. The cup is opened with each degree increase in temperature until the vapors ignite, and a flame is held at a sufficient height over the liquid surface. The acid value is determined by titrating the oil/fat directly against a standard potassium hydroxide/sodium hydroxide solution in an alcoholic medium. Cloud point and pour point were measured in accordance with ASTM standards as shown in Table 1. The sample was cooled in a glass tube and studied at 1 °C intervals until a foggy structure became visible under specified conditions. This temperature was denoted by the symbol CP. To determine the PP, the sample was cooled in a glass tube under controlled conditions and evaluated at 3 °C intervals until no movement was detected even when the surface was kept vertical for 65 s; the PP was then calculated to be 3 °C above the temperature at which the flow stopped. The elemental analysis of the WCO sample was performed using the Agilent 5110 vertical dual view (VDV) ICP-OES.

**Table 1:** Basic properties of this work WCO* and other referenced feedstocks.

Parameter	Current study (WCO)	Method	(WCO)_a_^40^	(WCO)_b_^41^	(WCO)_c_^43^	(Used sunflower oil)_d_^30, 44^	(Used palm oil)_e_^44^
Kinematic viscosity at 40 °C mm^2^/s	38	ASTM D-445	43.75	35.3	57.8	36	44.96
Density at 20 °C g/cm^3^	0.91	ASTM D-4052	0.96	–	0.92	0.92	0.92
Acidity mg KOH/g oil	5.20	ASTM D-3242	–	2.1	28.7	2.60	2.05
Iodine value gI_2_/100 g	75	ASTM D-5554	–	–	88.6	–	–
Pour point °C	− 10	ASTM D-97	–	–	–	− 5.15	12.3
Cloud point °C	− 5	ASTM D-2500	–	–	–	1	13
Flash point °C	198	ASTM D-93	200	–	–	183	164
Elemental analysis							
C wt. %	76.80		–		77.91	–	–
H wt.%	11.60		–		11.69	–	–
O wt. %	10.60		–		10.36	–	–
N wt.%	1		–		0.04	–	–
S wt.%	Nil		–		0	–	2.58
HHV*(MJ/Kg)	25.80		38.9			–	–
< C5 (%)	–		–	–	–	–	–
C5–C7 (%)	–		–	–	–	–	–
C8–C16 (%)	39.30		27.90	26.90	6.80	–	–
C17–C19 (%)	60.70		71.80	72.00	92.10	–	–
> C19 (%)	–		0.30	1.1	1.10	–	–

HHV* High heating value.

#### Characterization of catalyst

To characterize the prepared AL_2_O_4_ (Co/Zn) catalyst Nano particles, various analyses were performed. BET surface area, Barrett − Joyner − Halenda (BJH) pore size and pore volume have been measured using a Micromeritics model ASAP 2010 surface area analyzer with 99.9% purity nitrogen gas. The results were collected on a Tristar 3020 instrument. The conditions of degassing were at 400 °C with a heating ramp of 10 °C min-1 for 2 h prior to analysis. X-ray diffraction (XRD) patterns using Smart Lab Guidance and MDI Jade 8 instrument on a Rigaku RU2000 rotating anode power diffractometer (Rigaku Americas Corporation, TX) at a scan rate of 4° min-1 were used to identify the crystalline phases that present in the catalyst sample. High Resolution Transition Electron Microscopy (HRTEM) images have been gotten with a JEM 2010 HR TEM instrument, which is equipped with a digital camera system allowing the capture of both high-resolution images and electron diffraction patterns. The weight percentage of each element contained in the produced Co Zn /Al_2_O_4_ catalyst under investigation was assessed by Energy Dispersive X-Ray Analysis (EDX). This technique is based on hitting the examined specimen with electrons and creating a void inside the sample's atoms. This void is then filled by higher energy electrons from the atoms' outer shell. The transition of these higher energy electrons to lower energy shells resulted in the emission of some of their energy in the form of X-rays, the amount of which depended on the type of atom. As a result, each atom will have a distinct peak in the EDX spectrum with a specific height based on its concentration in the tested material. The SEM used was a "Quanta 250 FEG, Field Emission Gun" that was linked to a "Energy Dispersive X-ray Analyses, EDX Unit." Scanning the microstructure is accomplished by accelerating a finely focused electron beam at a potential difference of 30 kV. The specimen's microstructure was photographed at a magnification of 14 × up to 1,000,000.

FTIR analysis was performed to analyze the chemistry of the surface, especially different oxides in materials using Origin Jasco Infrared Spectrum (FT/IR-6100type A) over the wave number 4000–399 cm^−1^.

#### Bio jet blends characterization

The bio jet fuel fraction range obtained from the atmospheric distillation process of biofuel liquid product at the operating conditions of this study was blended in different weight percentages (5 percent, 10%, 15%, and 20%) with commercial jet A-1 and then characterized using the various ASTM-D7566 methods. The flash point, gum content, and freezing point were all measured as part of the characterization. Gum content is the residue of aircraft fuels, motor gasoline and other volatile distillates which cannot be evaporated. Fuel samples of 50 ml are evaporated under controlled conditions of temperature and the flow of air or steam (ASTM D7566).

**Figure 2:**
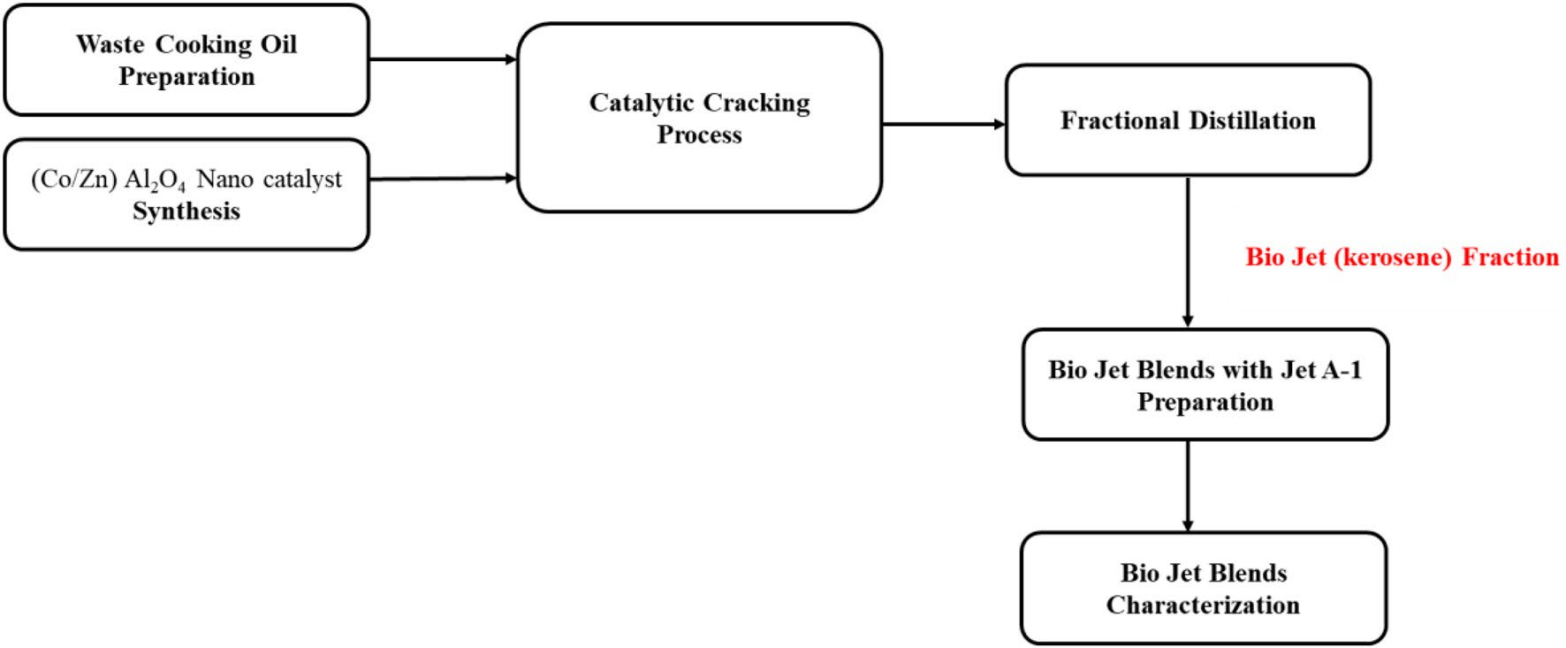
Work steps.

Standard ASTM are used to determine the freezing point of bio jet fuel samples. 15 ml of each sample is placed in a glass tube and frozen in liquid nitrogen until completely frozen. Reading the thermometer's indicator point yielded the freezing point. This happens when the temperature reading in the solid–liquid mixture stabilizes at the last thin crystal layer. Figure 2 shows the work steps in this study.

## Results and discussion

### Properties and composition of WCO

The composition and properties of WCO feedstock in this study are shown in Table 1. The oxygen content was estimated by the difference between the components, and the nitrogen content was very small (not exceeding 1% by weight) and the sulfur did not appear in the WCO. Therefore, fuels produced from WCO feedstocks are viewed as environmentally friendly green fuel. For the physical properties, the acidic value of WCO was 5.20 mg-KOH / g oil, which indicates that it contains a low percentage of free fatty acids. The iodine value of the oil was 75 g / 100 g of oil, indicating that the oil had many unsaturated bonds C = C. The viscosity was 38 mm^2^ / s at 40 °C and the density was at 20 °C 0.91 g / cm^3^. The Table 1 also lists the properties of a number of different types of other feedstock for oils that have been used in previous studies^30, 40–43^.

### Catalyst characterization

The prepared catalyst was characterized using BET, XRD, HRTEM, EDX, SEM and FTIR. Thus, the morphology, size and shape of the grains and pores could be verified. The main properties of the prepared catalyst depend on the micro-structure developed during processing and firing. It is affected by the main constituting phases, porosity and comparatively their shape, size and distribution. The results of the previous analyzes were presented and discussed below.

#### BET & N2 adsorption desorption isotherm

**Figure 3:**
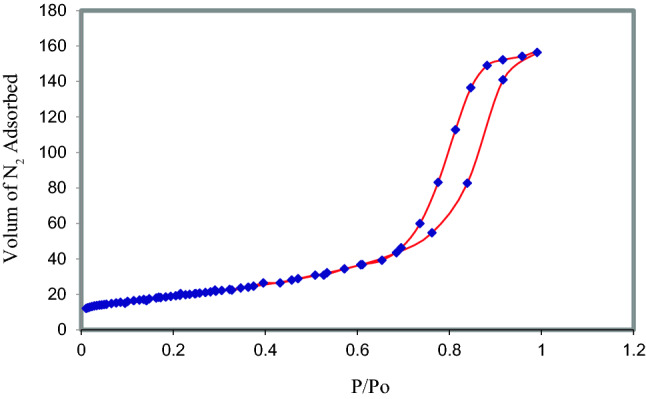
N2 adsorption desorption isotherm of Co/Zn-Al_2_O_4_ catalyst.

The nitrogen adsorption/desorption isotherms and pore size distributions of the various prepared samples are shown in Fig. 3. Three well distinguished regions of the adsorption isotherm are evident: (i) monolayer-multilayer adsorption, (ii) capillary condensation, and (iii) multilayer adsorption on the outer particle surfaces.

Apparently, the prepared sample have the type IV isotherm according to the classification of the International Union of Pure and Application Chemistry (IUPAC). Based on data in Table 2, it can be found that Co/Zn-Al_2_O_4_ catalyst have BET surface areas 69.2 m^2^/g^−1^, pore diameter 8.76 nm and pore volumes 0.22 cm3/g.

**Table 2:** Physicochemical properties of CoZnAl_2_O_4_ catalyst.

Surface area (m^2^/g)	69.20
Cross section area (Å^2^/molecule)	16.2
Pore diameter/nm	8.76
Pore size (cm^3^/g)	0.22

#### X-ray diffraction (XRD) analysis

**Figure 4:**
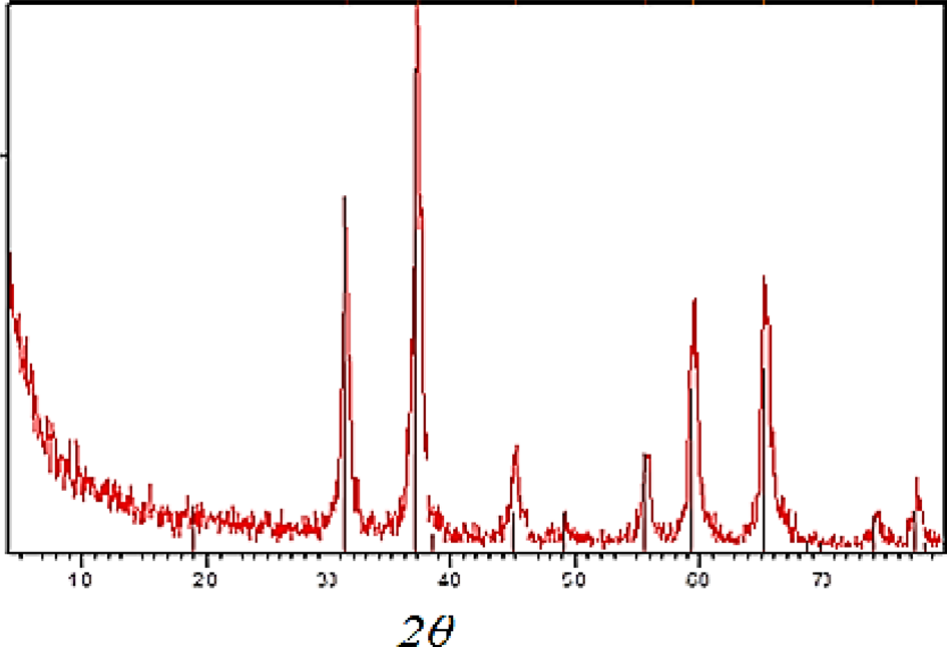
X-ray diffraction of Co/Zn-Al_2_O_4_ catalyst.

The powder X-ray diffraction patterns of prepared catalyst is shown in Fig. 4. In addition to the major phase of spinel Co/ZnAl2O4 structure (JCPDS card No. 05–0669), it can be seen that the sample crystallised in a single phase with a spinel structure and space group O7h and contains small amounts of ZnO (JCPDS card No. 36–1451) impurities^44, 45^ According to the literature. The observed diffraction peaks at 2 are 31.22, 36.77, 44.69, 48.98, 55.52, 59.27, 65.06, 73.97, and 77.12 and can be attributed to the (220), (311), (400), (331), (422), (511), (440), (620), and (533) planes of Co/ZnAl_2_O_4_ respectively. The mean grain size of the sample was calculated using the Scherrer formula based on the line broadening of the (220), (311), (511), and (440) peaks to be 13, 16, and 24 nm, respectively. According to the XRD results, the choice of aluminum salts has an effect on the phase purity of the final product.

#### High resolution transition electron microscopy (HRTEM)

**Figure 5:**
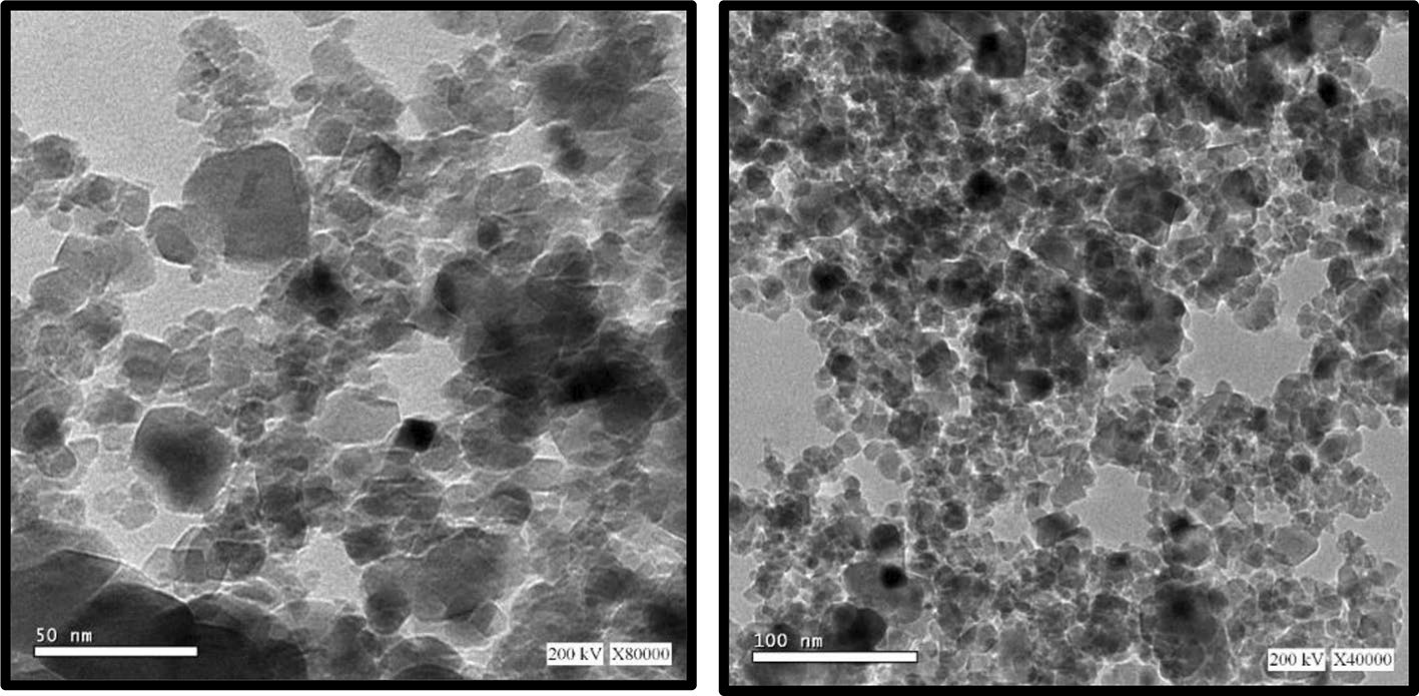
HRTEM of (Co/Zn) Al2O4 nano catalyst.

Figure 5 shows the transmission micrographs for the Co/Zn-Al_2_O_4_ sample. The image show that the zinc, cobalt aluminate particles are uniform and nanoaggregate; The zinc metal ions using Co^2+^ showed no significant changes in material morphology.

#### Energy dispersive X-ray analysis (EDX)

**Figure 6:**
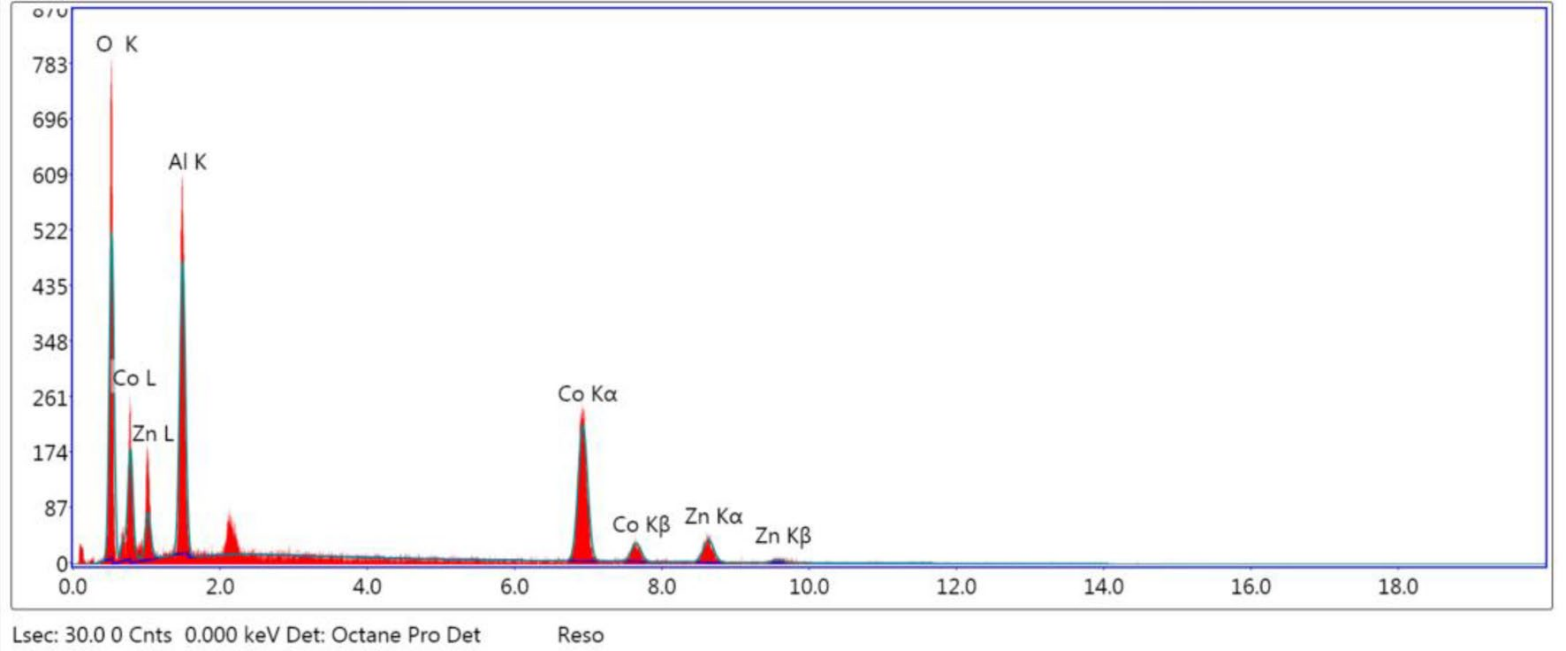
EDX spectrum of the parent Co/ZnAl2O4 catalyst.

The typical EDX spectra obtained for the parent Zn Co/Al_2_O_4_ catalyst are represented in Table 3 and Fig. 6.

**Table 3:** EDX analysis for Zn Co/Al_2_O_4_ catalyst.

Element	Weight %	Atomic %	Net Int	Error %
O	31.54	55.63	262.95	8.05
Al	21.42	22.4	286.51	8.34
Co	35.35	16.93	223.07	3.62
Zn	11.69	5.05	40.84	13.49

#### Scanning electron microscope (SEM)

**Figure 7:**
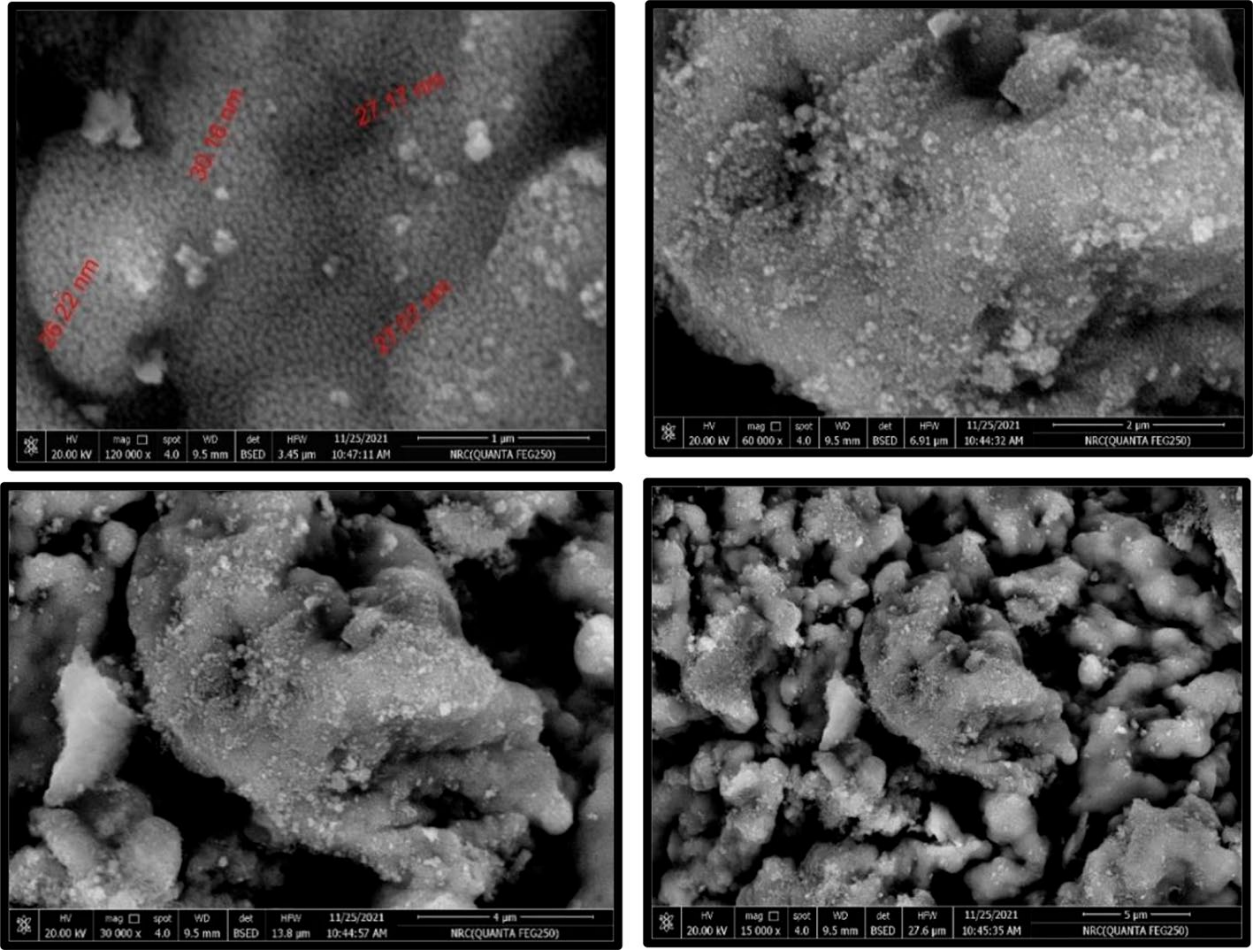
Scanning electron microscopy (SEM) images of CoZnAl2O4 catalysts.

The shape and surface morphology of CoZnAl_2_O_4_ nanoparticles were studied using HR-SEM. It was observed the HR-SEM pictures of pure and cobalt doped zinc aluminate nanoparticles homogeneous and grains are distributed uniformly as shown in Fig. 7. The SEM images show that the particle size ranges from 26.22 to 27.17 nm and 30.16 nm with little aggregation. Thus, the average particle size is approximately 28 nm.

#### Fourier transform infra-red spectroscopy (FTIR)

**Figure 8:**
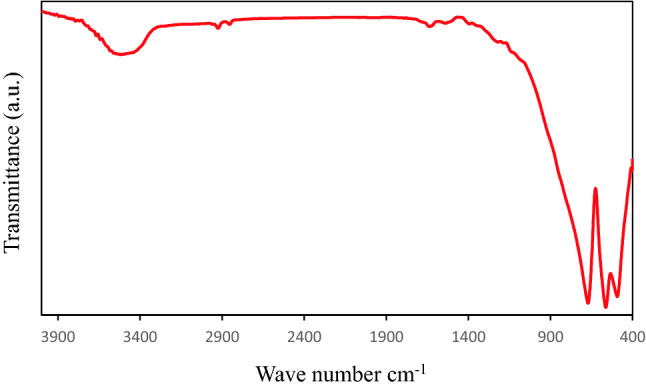
FT-IR of prepared Co/Zn-Al_2_O_4_ catalyst.

Fourier transform infrared (FT-IR) spectra of the Co/Zn-Al_2_O_4_ nanoparticles is shown in Fig. 8. The FT-IR spectra show a series of absorption peaks in the range of 400–4000 cm^−1^. The functional groups present in the samples can be deduced from the specific frequencies of the absorption peaks. Peaks at 1633, 656, 552, and 493 cm1 are present in the prepared sample and are attributed to the H–O-H bending vibration of adsorbed water^46, 47^, the Al-O symmetric stretching vibration (v1), the Al-O symmetric bending vibration (v2), and the Al-O asymmetric stretching vibration (v3), respectively^48^.

### The effect of cracking process temperature

Thermo-catalytic cracking process was conducted catalytically for 100 g of WCO for time of 15 min. using 2.5 g of (Co/Zn) Al_2_O_4_ catalyst. Experiments were carried out at five different temperatures to investigate the effect of temperature on the yield of bio fuel product, namely 300, 350, 400, 450, and 500 °C. The effect of cracking reaction temperature on biofuel yield is shown in Table 4. According to results of this Table, the cracking temperatures have a significant influence on the biofuel product as is an important key parameter in the catalytic cracking of vegetable oil^41^.

**Table 4:** Results of temperature effect on thermo catalytic cracking process*.

Temperature °C	Liquid product yield (%)	Residue (%)	Gas product (%)
300	18	72	10
350	56.40	14.10	29.50
400	65.25	7.25	27.50
450	67	3.50	29.50
500	67.60	3.40	29

WCO Feed = 100 g, Reaction Time = 15 min, Catalyst Load = 2.5 g, Initial Pressure of Nitrogen = 5 bar.

From the results shown, it appears that the higher the temperature, the greater the amount of liquid obtained from the thermo catalytic cracking process.

With increase in temperature from 300,350,400 to 450 °C the yield of liquid product % weight gradually increased from 18%,56.4%, 65.25% to 67% respectively.

The higher reaction rate at 400–450 °C temperature can be believed to be due to the opening of the catalyst sites, which results in more available active sites of the catalyst, resulting in a higher liquid biofuel yield. However, increasing the temperature further did not significantly increase the yield, whereas increasing the temperature to 500 °C resulted in a very slight increase in yield. This is because further temperature increases induced secondary cracking, which favors gas yield^41, 48^. Thus, the reaction mixture temperature of 450 °C was proposed to be economically suitable for further experimentation, yielding approximately 67 percent liquid biofuel in 15 min. Carbon dioxide, carbon monoxides, and hydrocarbon compounds C1-C5 make up the resultant gas^49^. Although its proportion may have reached close to 30%, the current study focused on the liquid product and the prospect of employing it as a bio jet fuel. The residue % of the crude biofuel product was calculated from the collected fraction of the atmospheric distillation process above 360 °C.

### The effect of cracking process time

The effect of reaction time on biofuel liquid product yield was investigated over three-time intervals of 10, 15, and 30 min.

The results in Table 5 show that increasing the reaction time had a direct effect on the total yield% of gas and liquid biofuel products produced by the thermo-catalytic cracking process. As of 15 min, the total yield was 96.5 percent, while it was 92.5 percent at 10 min. The catalytic cracking rate was higher up to 15 min, which could explain this.

**Table 5:** Results of cracking time effect on thermo catalytic cracking process*.

Time min	Liquid product yield (%)	Residue (%)	Gas product (%)
10	67.50	7.50	25
15	67	3.50	29.50
30	65.5	3.45	31

WCO Feed = 100 g, T = 450 °C, Catalyst Load = 2.5 g, Initial Pressure of Nitrogen = 5 bar.

Increased reaction time up to 30 min had no effect on liquid biofuel yield, with a slight decrease. This could be because the prolonged contact time of the liquid product with the reaction mixture resulted in the conversion of side products^41^.

As a result of the preceding data, it is possible to conclude that an acceptable yield was obtained from the process thermo-catalytic cracking in just 15 min. Furthermore, according to this study, (15 min.) was considered the optimal time of cracking process in terms of economics and energy saving.

### The effect of catalyst load on the cracking process

The selection of catalyst is an important factor in thermo-catalytic cracking processes due to its role in product yield and component selectivity for biofuel. The efficiency of a catalyst for catalytic cracking is determined by its surface area, pore size, pore volume, and active site^41, 50^. The effect of catalyst concentration on liquid biofuel yield was investigated using a thermo-catalytic cracking reaction of WCO at catalyst loadings of 1.5, 2, and 2.5 g. under constant conditions of 450° C and 15 min cracking time and an initial nitrogen pressure of 5 bar. Table 6 shows the effect of (Co/Zn) Al_2_O_4_ Nano catalyst loading on yield% of biofuel. It was observed that the total yield% of biofuel products increased as catalyst loading increased where it was 96% at 1.5 g catalyst loading and slightly increased to 97.35% at catalyst loading of 2 g. This is obvious because the rate of catalytic cracking reaction is directly proportional to the number of active sites on the catalyst, so higher catalyst loading will provide more active sites for the reaction. This is true up to a certain catalyst loading, after which the catalyst loading no longer serves as a limiting factor. When the catalyst loading was increased to 2.5 g, the total yield percent and liquid biofuel yield decreased. This is due to the fact that increasing the heterogeneous catalyst loading resulted in aggregation of catalyst active sites, reducing catalytic activity. As a result, a large amount of residual coke was deposited on the catalyst active sites, resulting in catalyst deactivation. This coke decreases surface area of the catalyst which leads to decrease in active site or it may function as a toxin and impair catalytic activity. These results are in analogous to the results reported in literatures^43, 51, 52^.

**Table 6:** Results of catalyst amount effect on thermo catalytic cracking process*.

Catalyst load (g)	Liquid product yield (%)	Gas product (%)	Residue (%)	Total yield* (%)
1.5	76	20	4	96
2	69.35	28	3.65	97.35
2.5	65.25	27.50	7.25	92.75

*Sum of liquid product & gas product only.

WCO Feed = 100 g, T = 450 °C, Time = 15 min., Initial Pressure of Nitrogen = 5 bar.

**Figure 9:**
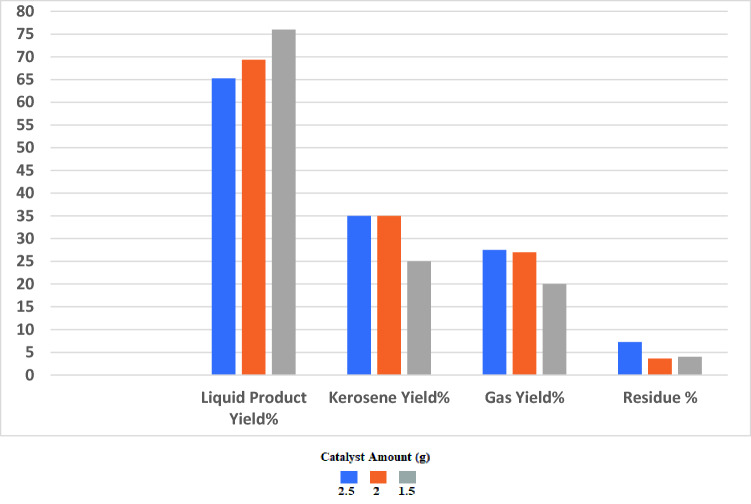
Distribution of the products from the cracking of WCO at 450 °C on the ZnCoAl_2_O_4_ nano catalyst with wt. %: 2.5,2 and 1.5.

Thus, the criterion in choosing the optimal amount of it is to determine the highest bio jet fracture yield (the jet range fraction) and refer to Fig. 9 which shows the results of the fractional distillation process for experiments in which the three different quantities were used in the thermo-catalytic cracking process. The of bio jet (kerosene) yield % was 35% when the amount of catalyst was 2.5 g and 2 g while the yield % of bio jet range product was just 25% when using 1.5 g of it. From the previous data and from the economic point of view it can be concluded that the optimum quantity of the prepared (Co/Zn) Al_2_O_4_ Nano catalyst in this study was 2 g, which represents 2% of the WCO feedstock.

### Results of bio jet fraction range blends with Jet-A

After a fractional distillation stage, three blend samples of biofuel products were withdrawn in the ranges (140–280° C), (150–260° C), and (150–240° C), and each sample was blended with 95% Bio Jet-A. Following that, tests were performed to ensure that the properties of these binary mixtures meet ASTM-D7566 jet fuel specifications. The properties of these blends are shown in Table 7. It should be noted that when a wider range of distillates was used (140–280 °C), the properties of the resulting biofuel were outside of the ASTM-D7566 specifications, whereas when a narrower range of distillates was used (150–260 °C), the flash point value and gum content improved. While the freezing point value remained out of the ASTM-D7566 specification. However, the properties were improved when the distillate products were used in a more specific range of (150–240 °C), where the flash point was (41 °C), gum content was (2 mg/100 ml), and freezing point was (-55 °C), all of which met the international ASTM- D7566 specification of bio-jet fuel in that fraction of distillation range.

**Table 7:** Results of physical properties of binary jet fuel A-1 blends with samples of distillate product.

Test	Jet A-1	ASTM D7566-	Blend (1)	Blend (2)	Blend (3)
Flash point °C	44	38 min	32	41	41
Gum content mg/100 ml	1.20	7 max	3	1.80	2
Freezing point °C	− 54	− 47 min	− 20	− 24	− 55

*Blend (1): 5% Distillate product sample (95%) in range (140–280° C) + 95% Bio-jet A-1.

Blend (2): 5% Distillate product sample (95%) in range (150–260° C) + 95% Bio-jet A-1.

Blend (3): 5% Distillate product sample (95%) in range (150–240° C) + 95% Bio-jet A-1.

From the foregoing, it can be concluded that the optimal cut-off from the distillation process, which represents a fraction of bio-jet fuel was in the range from 150 to 240° C, which had the best properties. These findings are comparable to those reported in the literature^53–55^.

Four bio-jet fuel blends (5 percent, 10%, 15%, and 20% by weight) were prepared by adding with commercial jet A-1 fuel and then testing some physical properties.

**Figure 10:**
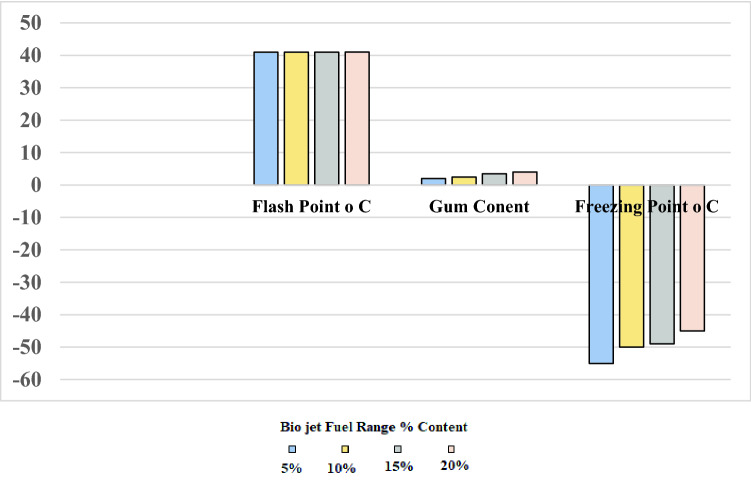
Physical properties of binary jet A-1 blends with different ratios of bio jet fuel product range and jet A-1.

The outcomes are depicted in Fig. 10 which demonstrated that the flash point values of the four blends (41 °C) did not change. While increasing the percentage of bio jet fuel in the blend resulted in an increase in both gum content and freezing point as follows: gum content 2, 2.5, 3, and 4 mg/100 ml and freezing point values of − 55, − 50, − 49, and − 45 °C for the blends of 5%, 10%, 15%, and 20%, respectively.

## Conclusion

- The analysis revealed that the prepared catalyst had an average surface area of 69.20 m^2^/g, a cross-sectional area of 16.2 m^2^/molecule, an average particle size of approximately 28 nm, and a pore size of 0.22 cm^3^/g.
- The optimum conditions for the thermo catalytic cracking of WCO on prepared zinc (Co/Zn) Al_2_O_4_ nanoparticles were:450 °C, 2% weight % of the catalyst and 15 min. reaction time.
- Bio-jet fuel fraction was obtained by atmospheric fractional distillation of the crude liquid bio fuel produced from the catalytic-cracking process at temperature ranging from 150 to 240 °C, and it had fairly acceptable physical properties and met the jet fuel ASTM specifications.
- According to the results of this study, thermo-catalytic cracking of WCO produced a convenient freezing point (-55 °C) of a binary sample of 5% bio jet distillate product with 95% jet A-1.
- WCO bio-jet production is advantageous for bio-jet fuel production due to its low cost and alternative waste utilization to reduce environmental harm.

## Data Availability

All data generated and analyzed during this study were included in this published article.
